# The capacity and organization of gustatory working memory

**DOI:** 10.1038/s41598-022-12005-x

**Published:** 2022-05-16

**Authors:** Shirley Xue Li Lim, Richard Höchenberger, Iryna Ruda, Gereon R. Fink, Shivakumar Viswanathan, Kathrin Ohla

**Affiliations:** 1 Cognitive Neuroscience (INM-3), Institute of Neuroscience and Medicine, Forschungszentrum Jülich, Jülich, Germany; 2 Department of Neurology, University Hospital Cologne, and Medical Faculty, University of Cologne, Cologne, Germany

**Keywords:** Psychology, Human behaviour

## Abstract

Remembering a particular taste is crucial in food intake and associative learning. We investigated whether taste can be dynamically encoded, maintained, and retrieved on short time scales consistent with working memory (WM). We use novel single and multi-item taste recognition tasks to show that a single taste can be reliably recognized despite repeated oro-sensory interference suggesting active and resilient maintenance (Experiment 1, N = 21). When multiple tastes were presented (Experiment 2, N = 20), the resolution with which these were maintained depended on their serial position, and recognition was reliable for up to three tastes suggesting a limited capacity of gustatory WM. Lastly, stimulus similarity impaired recognition with increasing set size, which seemed to mask the awareness of capacity limitations. Together, the results advocate a hybrid model of gustatory WM with a limited number of slots where items are stored with varying precision.

## Introduction

Working memory (WM) is the faculty of actively storing information for short periods^1,2^. Since its original formulation, the organization of WM and its neural substrate have been subject to extensive investigation^3–5^. However, research into the generality of WM for different kinds of information^6,7^ has neglected an essential chemical sense: the sense of taste. This is surprising given the relevance of taste information processing to identify nutrients and maintain homeostatic balance^8,9^. Furthermore, the taste of previously consumed food substances guides future dietary decisions. As this memory for prior tastes is essential for adaptive behavior, long-term gustatory memory, particularly conditioned taste-aversion, has been the focus of numerous studies^10–14^. However, the possibility that multiple recently encountered tastes (such as sweet or sour) could be stored and maintained in WM has received almost no consideration, except for one study^15^. This omission is even more notable given that working memory for smell and flavor has been the focus of previous animal^16^ and human^17,18^ studies. Consequently, to what extent information about multiple tastes can be actively maintained in WM and its storage organization remains elusive.

The current study sought to investigate gustatory WM by assessing its storage *capacity*. Due to the methodological challenges of delivering multiple taste stimuli in a precisely controlled sequential manner, we operationalized gustatory WM’s storage capacity as the maximum number of unique tastes (items) that could be discretely maintained to enable subsequent recognition. In other sensory modalities, the study of WM’s storage capacity has provided valuable insights into its organization—for example, as being distributed resource-based^19–21^ or fixed slot-based^22,23^. However, relating the capacity limits for taste information to WM organization is challenging due to several considerations specific to the gustatory system’s mostly peripheral organization.

**Figure 1:**
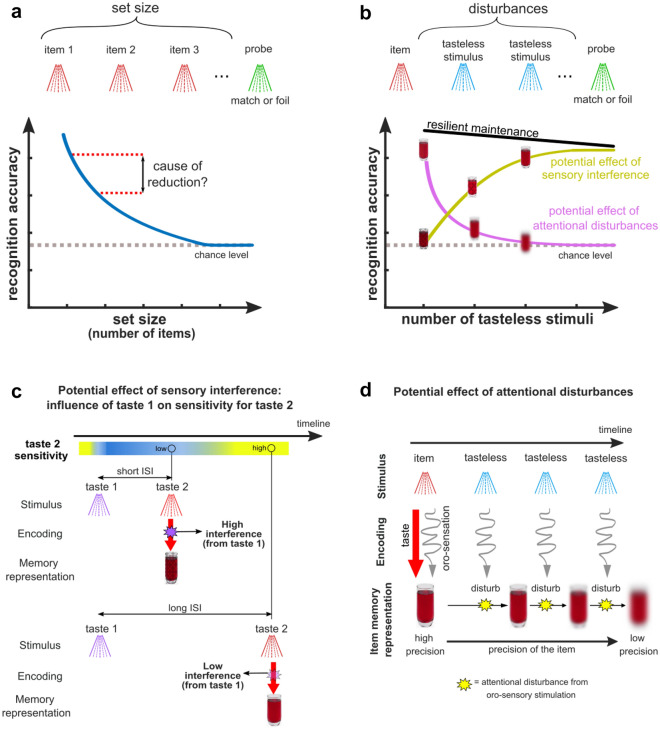
Illustration of hypothetical outcomes and inference effects in estimating taste WM capacity. (**a**) A multi-item recognition task (upper row, Experiment 2) requires a judgment whether the probe taste corresponds to one of the previously encountered taste items (match) or not (foil). The lower panel illustrates the typically expected accuracy decrease with increasing set size. (**b**) In a single-item recognition task (Experiment 1), a set consists of a single item with following items replaced by tasteless stimuli (disturbances). The lower panel illustrates predicted accuracy changes with increasing disturbances based on alternative hypothesized mechanisms. *Sensory interference* (also see panel **c**) predicts increased accuracy with an increasing number of rinses and time between the item and probe. *Attentional disturbance* (also see panel **d**) conversely predicts a steep decrease in accuracy with increasing disturbances and time. *Resilient maintenance* predicts accuracy that is relatively unaffected by disturbances and time. (**c**) Adaptation in response to any item could affect the sensory encoding of the subsequent item(s). Impaired sensory encoding would mirror WM performance. Adaptation effects would be weaker as interstimulus intervals (ISI) increase. (**d**) Assuming that the oro-sensory stimulation is a “disturbance”, this “disturbance” could reduce the fidelity of the item’s representation in WM.

A general view of a limited WM capacity is that it explains why recognition accuracy decreases with increasing set size^2,21,24^ (Fig. 1a). However, decreases in recognition accuracy can be influenced by factors unrelated to gustatory WM, such as irrelevant cross-modal information that accompany sensory experiences. This constitutes a particular concern for gustation, which is inherently coupled with non-gustatory sensations. For instance, when eating an apple, its appearance, smell, texture, temperature, even sound when taking a bite are inextricably linked with its taste, see^25^. These factors can affect recognition processes and distort capacity estimates of gustatory WM. Additionally, the possible physical mixing of sequentially presented taste stimuli on the tongue as well as the relative proneness of the gustatory system to quick sensory adaptation^26^ are potential sources of sensory interference that might reduce the fidelity with which tastes are encoded in WM (Fig. 1c).

To limit the role of cross-modal information, all stimuli in our study were odorless and colorless liquids with a similar viscosity presented at a constant temperature. The concern about mixing tastes was countered by including a tasteless stimulus as a rinse between stimuli to remove residual tastes. However, tastes are unavoidably associated with an oro-somatosensory touch due to contact with the tongue, and these touch stimulations might present attentional “disturbances” that could degrade WM performance^7,27–29^. Based on these considerations, we investigated the organization of gustatory WM in two experiments. To obtain a baseline measure of the magnitude of critical non-capacity related factors, i.e., sensory interference and attentional distraction, in Experiment 1, we evaluated recognition of a single taste item in the presence of a variable number of oro-sensory disturbances presented between item and probe. The opposing hypothesized effects of sensory and attentional interference (see Fig. 1b) permit us to assess their contribution to gustatory WM, as reductions in sensory interference with time would be predicted to increase taste recognition accuracy (Fig. 1c). In contrast, increases in attentional disturbances would be predicted to cause a marked drop (Fig. 1d). In Experiment 2, we sought to estimate the capacity of taste working memory. For this, we used a list memory task where participants were to remember sets of distinct sequentially presented tastes presented in various set sizes to estimate the unknown capacity of gustatory WM. Since recognition accuracy can be modulated by stimulus-similarity effects^30^, we sought to account for the role of these effects when estimating capacity.

## Methods

### Participants

Participants were recruited at the Research Center Jülich, Germany. The sample size was based on a priori power calculations using G*Power 3.1^54^. Assuming a power of 0.90 and a medium effect size of f = 0.3 in an rmANOVA a sample of N = 19 (1-factorial rmANOVA with 5 levels) would be sufficient. Twenty-one healthy adults (15 females; age M = 28.43, SD = 3.9, range: 22–42 years) participated in Experiment 1 and twenty healthy adults (9 females; age M = 26.6; SD = 3.31, range: 22–33 years) participated in Experiment 2. In Experiment 2, one participant did not complete the study because they failed to detect all bitter taste trials in the taste detection task. Participants were generally healthy, normal weighted (Body Mass Index < 28), had no current or past neurological disorders or taste disturbances, and did not take medication known to influence brain function or perception (self-report). Female participants reported not being pregnant. Total participation time was 6 h (Experiment 1) and 7 h (Experiment 2) distributed over 2 and 3 sessions, respectively. All participants signed an informed consent form before participation and received monetary compensation. The study was approved by the ethical review board of the German Psychological Society (DGPs) and complied with the revised declaration of Helsinki.

### Stimuli

Stimuli were based on four aqueous solutions prototypical for the basic tastes: sweet, salty, sour, and bitter. The fifth basic taste quality, umami, was not used because it requires familiarity for a perceptually salient experience and is perceptually associated with salty and sweet^55^. The basic tastes were produced by filling up 190 g sucrose (food quality), 30 g sodium chloride (food quality), 9 g citric acid (CAS 77-92-9), and 0.25 g quinine hemisulfate salt monohydrate (CAS 207671-44-1) with distilled water to 1L, respectively. The taste solutions were then mixed at a 1:1 ratio to achieve six binary mixtures: sweet–salty, sweet–sour, sweet–bitter, salty–sour, salty–bitter, sour–bitter, resulting in ten different taste solutions. Artificial saliva was prepared by filling up 0.92 g sodium bicarbonate (Euro OTC Pharma GmbH, Article Number: 178900) and 0.105 g potassium chloride (Euro OTC Pharma GmbH, Article Number: 162070) to 1L with distilled water. Artificial saliva served as a rinse after the tastants and as non-gustatory, oro-somatosensory distraction because it is tasteless^56^. All solutions were cooled at 4 °C when not used and renewed every 72 h.

### Apparatus

Stimuli were delivered with a high-precision computer-controlled syringe pump system^39^. We used five syringes for each of the four basic tastants and one for artificial saliva. Each syringe was connected to an inlet tubing that supplied the syringe with the tastant from a glass reservoir and an outlet tubing, which delivered the tastants to the participants. Each outlet was mounted in a manifold with five inlets (one for each solution) and one outlet with a 1–2 mm stub placed on participants’ tongues. Two syringes delivered stimuli with 7 ml volume at a total flow rate of 3.5 ml/s.

The experiment was controlled by PsychoPy version 3.0.6^57^ running on Windows 10. Responses were recorded using a three-button response box (The Black Box Toolkit, Sheffield, UK) that was comfortably placed in front of participants.

### Experimental paradigm and procedure

Both experiments consisted of a taste detection and evaluation (TDE) task and a taste recognition task with a single item in Experiment 1 and multiple items in Experiment 2. Experiments 1 and 2 consisted of two and three sessions, respectively, on separate days no more than 14 days apart. Participants performed the TDE task before the taste recognition task.

#### Taste detection and evaluation (TDE) task

The TDE task served to test whether participants could taste all stimuli. For this, we presented the 10 tastants (repeated four times) and artificial saliva (repeated 12 times) resulting in 52 trials. Each trial began with the visual cue “Tongue out”. This cue was presented for 0.75 s on the screen, followed by a 3-2-1-countdown (from 3-2- to 1) over 1.5 s. Next, a central fixation cross appeared on the screen concurrently with stimulus delivery for 0.75 s. After the stimulus presentation, participants were prompted to indicate whether they tasted anything by pressing the response box’s left or right button to answer yes or no. Participants were then cued to rate the intensity and pleasantness of the stimulus by moving an arrow with the buttons along on horizontal 11-point scales anchored with “Not at all” and “Very intense” for intensity and with “Unpleasant” and “Very pleasant” for pleasantness. The trial ended with a presentation of artificial saliva (tasteless stimulus) for 2 s to remove any residual tastant before the subsequent trial, which started after a varying inter-trial interval (ITI) of 10–15 s.

#### Single-item recognition task (Experiment 1)

**Figure 2:**
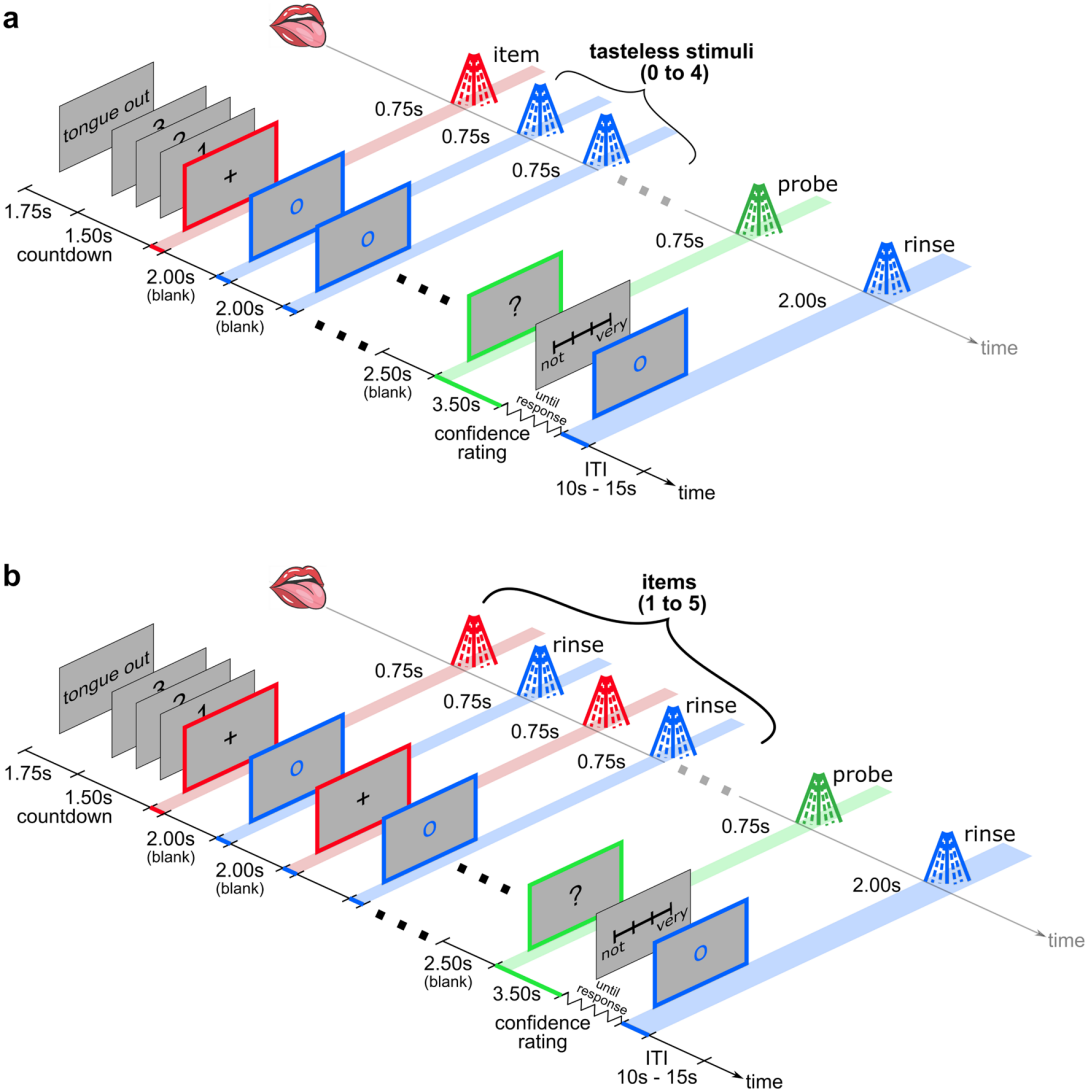
Procedure of a single trial. (**a**) In Experiment 1 (Single Item Recognition), participants received an item and a probe tastant that could be identical (match) or different (foil). Item and probe were interleaved with zero to five tasteless stimuli that served as a cross-modal disturbance. Participants were to indicate by button press whether the item and probe matched. (**b**) In Experiment 2 (Multi-Item Recognition), one to five items were sequentially presented. A tasteless stimulus (rinse) separated the items. Participants were to indicate whether the probe matches any of the items in the set. In both experiments, participants rated the confidence of their decision on a 4-point numeric scale (1 = not confident, 4 = very confident). Colored lines on the timeline represent the duration of the visual display. Shades represent the duration of the spray. Taste stimuli are shown in red, the tasteless rinse in blue, and the probe in green. Colors are not displayed in the experiment and are only shown for clarity.

In the single-item taste recognition (SIR) task (Fig. 2a), each trial began with presenting a single taste item that participants had to remember. This taste item was followed by tasteless artificial saliva stimuli that simulated the task-irrelevant oro-sensory disturbances typically accompanying any taste presentation. The trial ended with the presentation of a second tastant (probe). Participants indicated whether the probe was identical to (match) or different from (foil) the first taste item, followed by a rating of their confidence in this decision. To limit expectancy effects, the number of disturbances (1–5) on each trial was unknown to participants and, on one-sixth of trials, the probe was presented directly after the item without intervening disturbance stimuli (Fig. 1a).

Like the TDE task, each trial began with a cue “Tongue out” and a countdown. Next, simultaneously with a central black fixation cross presentation, participants received a tastant for 0.75 s followed by a blank screen of fixed inter-stimulus-interval (ISI) of 2 s. On trials with at least one disturbance, one or more tasteless stimuli were presented next, together with a central blue fixation circle, for 0.75 s followed by a 2 s ITI. After the last stimulus on a trial and an additional 0.5 s delay, the probe was presented for 0.75 s together with a question mark on the screen. Participants had 3.5 s to respond by button press whether item and probe were the “same” or “different” from probe delivery on. They were then asked to rate their confidence at the time of response by moving an arrow along a 4-point scale anchored with “Not at all” (left) to “Very” (right). The trials ended with a tasteless stimulus presented for 2 s, and the next trial started after a varying ITI of 10–15 s. Overall, the task comprised 400 trials with match and foil evenly distributed over five different numbers of disturbances.

#### Multi-Item recognition task (Experiment 2)

In the multi-item recognition (MIR) task (Fig. 2b), participants were to remember one to five sequentially presented tastes (items) on each trial, representing set sizes of one to five (referred to as S1 to S5), respectively. A tasteless stimulus (rinse) followed each item to cleanse sapid residues with minimal taste recognition disturbance to minimize sensory interference. Item memory was then tested with a probe stimulus. Participants indicated whether the probe matched any of the items in the remembered set (match) or not (foil) and they then rated confidence in that decision.

The procedure was similar to Experiment 1 with the difference that (1) sets were composed of 1–5 tastants (items), and (2) items were interleaved with a brief tasteless stimulus to rinse for 0.75 s. Each of the five set sizes consisted of 40 match and 40 foil trials resulting in 400 trials.

#### Procedure

During each experiment, participants were seated in an acoustically shielded chamber (Studiobox GmbH, Walzbachtal, Germany), and the gustometer and computers were placed outside to minimize any auditory distractions. Additionally, participants listened to ocean waves (Spotify) through insulated in-ear headphones to minimize auditory cues from the gustometer’s valves switching. Participants sat in front of a 22 inch LCD monitor at 55 cm distance with their forehead comfortably resting on a headrest, which also holds the manifold and nozzle in place. During the experiments, participants placed their tongue’s tip on the outlet nozzle upon displaying a visual cue. They were to remain in this position until the end of a given trial, indicated on the screen. Participants were allowed to take a sip of water and rinse their mouths during the ITI.

#### Trial design

All trial types and conditions were presented in pseudo-random order. In order to minimize potential taste-specific effects WM, i.e., sweet may be more memorable than bitter, we rigorously controlled for taste-specific effects in our randomization of Experiment 2, which applied the following constraints: no repetition of tastants within a set/trial, no repetition of the same set of tastants (irrespective of the order of tastants), no repetition of the target tastant for each serial position and set size (providing an equal distribution of stimuli across positions in all set sizes), uniform distribution of the probes across stimulus space, and subsequent trials shared no more than 2/5 of tastants. Lastly, response buttons were counterbalanced across participants and sessions.

### Statistical analysis

Data were analyzed with JASP version 0.12.2^58^. Trials without a response were excluded (0.6% in Experiment 1 and 1.2% in Experiment 2). Data were analyzed using rmANOVAs, simple main effects ANOVAs, and paired sample *t* test. A Greenhouse–Geisser correction was applied in case of violations of sphericity as identified by Mauchly’s test. Bonferroni correction was applied to all post hoc pairwise comparisons (two-tailed Student *t* test). Corrected p-values and confidence intervals were reported; Cohen’s d does not correct for multiple comparisons. The ⍺ level was set to 0.05.

**Figure 3:**
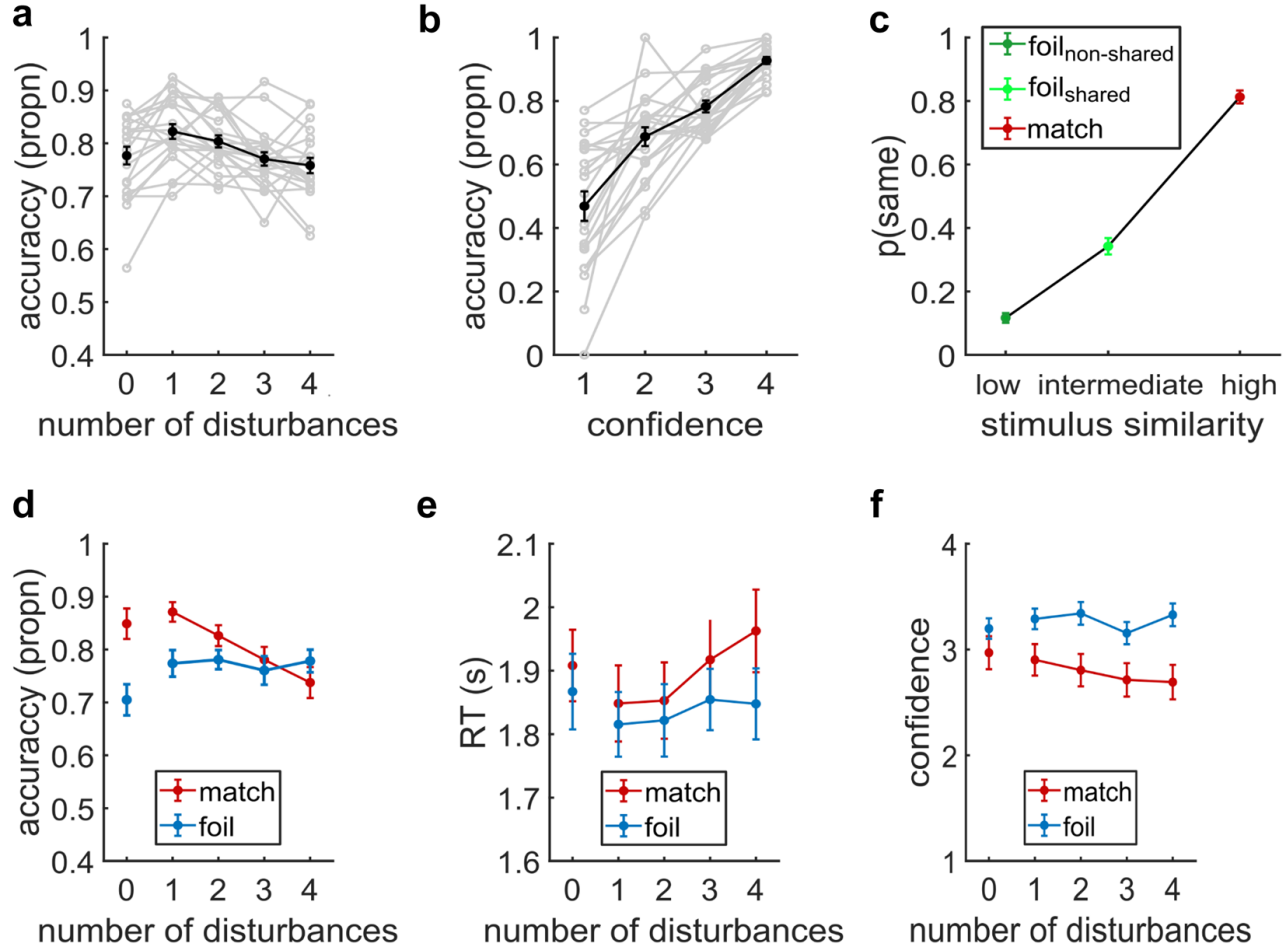
Experiment 1. (**a**) Recognition memory accuracy (proportion correct, propn) across the number of disturbances. (**b**) Accuracy for each confidence rating (1 = not confident, 4 = very confident). (**c**) Proportion of same responses for item probe pairs with different levels of stimulus similarity: highest in similarity (match), intermediate in similarity (foil pairs, shared component), lowest in similarity (foil pairs, non-shared component).  (**d**) Recognition memory accuracy for match and foil trials across the number of disturbances. (**e**) RT (in seconds) averaged across the number of disturbances, separately for match and foil trials. (**f**) Confidence averaged across the number of disturbances, separately for match and foil trials. Data points are means, error bars are SEM. Gray lines represent single-subject data (N = 21).

In Experiment 1, we used a one-way rmANOVA with the factor *number of disturbances* (1–4) for recognition accuracy (Fig. 3a); the factor *similarity* (low, intermediate, high) for the proportion of “same” responses (p(same); Fig. 3c); and the factor *confidence* (1–4) for recognition accuracy (Fig. 3b). To test whether match and foil trials differed, we used separate two-way rmANOVAs with the factors *number of disturbances* (1–4) and *trial type* (match, foil) for recognition accuracy (Fig. 3d), RTs (Fig. 3e), and confidence (Fig. 3f). Significant interactions were followed up with simple main effect analyses if applicable.

**Figure 4:**
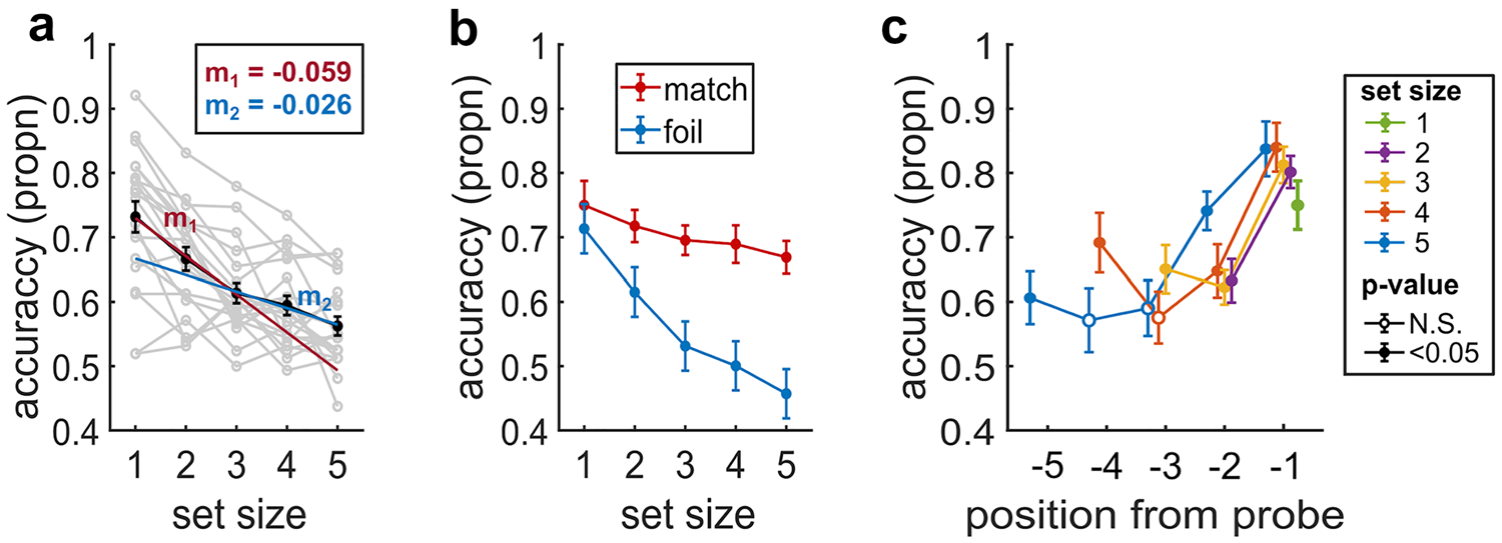
Recognition accuracy in Experiment 2. (**a**) Recognition accuracy across set sizes. The red line is fitted to the first three data points with a slope (m_1_) of − 0.059, and the blue line is fitted to the last three data points with a slope (m_2_) of − 0.026. (**b**) Recognition accuracy of match and foil trials across set size. (**c**) Serial positions for match trials. Recognition accuracy for each item in a given set size relative to the probe. I.e., in set size 5, the first item presented is at position − 5 from the probe. Accuracy is the proportion of correct responses (propn). Data points are means, error bars are SEM. Gray lines represent single-subject data (N = 20). Filled circles represent accuracy significantly above chance (p < 0.05); open circles represent accuracy not significantly above chance.

**Figure 5:**
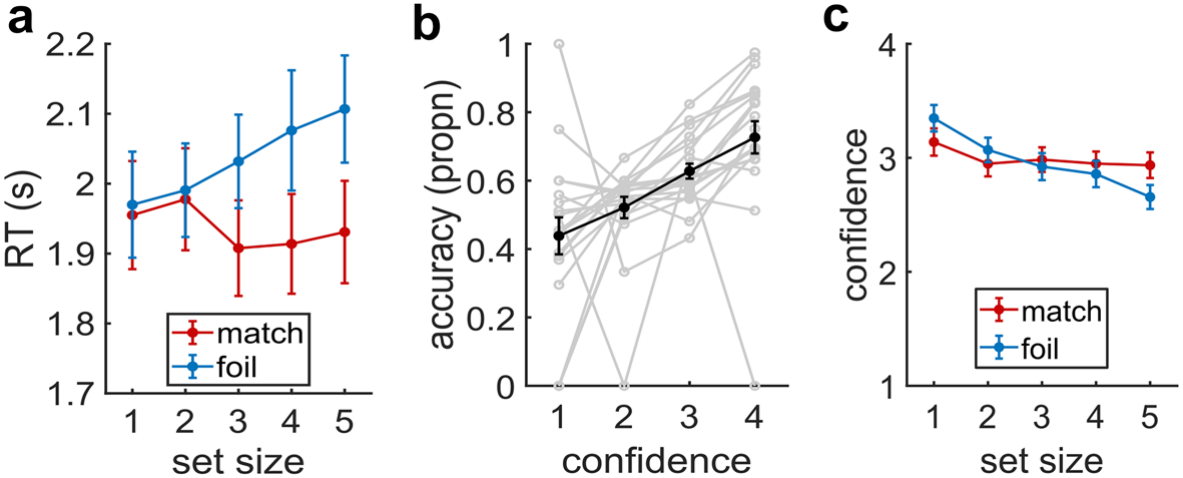
Response times and confidences in Experiment 2. (**a**) Response times (in seconds) for match and foil trials across the set size. (**b**) Recognition accuracy (in proportion correct) for each confidence level (1 = not confident, 4 = very confidence). (**c**) Confidence for match and foil trials across the set size. Data points are means; error bars are SEM. Gray lines represent single-subject data (N = 20).

In Experiment 2, separate one-way rmANOVA with the factor *set size* (1–5; Fig. 4a) and *confidence* (Fig. 5b) were performed for recognition accuracy. To test whether match and foil trials differed, two-way rmANOVAs with the factors *set size* (1–5) and *trial type* (match, foil) were performed for recognition accuracy (Fig. 4b), RTs (Fig. 5a) and confidence (Fig. 5c). To test for the serial-position effects on recognition accuracy (Fig. 4c), we performed separate one-way rmANOVAs for set size 5 (5 levels), 4 (4 levels), and 3 (3 levels). Lastly, we performed a paired *t* test by testing an alternative hypothesis of μ > 0.5, to see if each position in a set size is significantly higher than chance level.

## Results

### Stimulus validation

Due to the large inter-individual variability in taste perception^31,32^, it was critical to validate that participants tasted the stimuli. Therefore, we used a taste detection and evaluation task to confirm that participants could perceive the tastants before participating in the taste memory experiments. For these, participants were presented with taste and tasteless stimuli in a pseudo-randomized order. Participants indicated whether they tasted something or not by button press, and detected tastes were further rated in terms of intensity and pleasantness. Taste detection accuracy was high in both experiments (Experiment 1: M = 97.5%, SD = 6%; Experiment 2: M = 98.5%, SD = 4%; Suppl. Fig. 1). Of the ten tastes, bitter had the lowest detection accuracy (Experiment 1: M = 87%; Experiment 2: M = 88.8%), consistent with the known variability in bitter taste perception^32^. All tastes were rated as intermediately intense (Experiment 1: M = 6.52, SD = 1.61; Experiment 2: M = 6.74, SD = 1.42) and moderately pleasant (Experiment 1: M = 4.58, SD = 1.51, Experiment 2: M = 4.50, SD = 1.49; Suppl. Fig. 1). The apparently high consistency in taste detection accuracy and intensity and pleasantness ratings in the two experiments demonstrated that the stimuli were reliably perceived across participants.

### Baseline measure of interference effects (Experiment 1)

The objective of Experiment 1 was to obtain a baseline measure of critical non-capacity-related factors that might nevertheless affect recognition and confound estimates of capacity. For this purpose, we tested the resilience of a single taste memory to non-taste disturbances using a SIR task (Figs. 1 and 2b). If the single item was resiliently maintained in WM, recognition accuracy should be similar across different numbers of oro-sensory disturbances. In contrast, if the item’s representation was affected by attentional distraction (e.g., due to bottom-up shifts of attention produced by oro-sensory stimulation) or sensory interference (e.g., due to a loss of sensitivity produced by prior tastes), accuracy should markedly change with an increasing number of tasteless stimuli between the single item and the probe stimulus (Fig. 2b–d).

#### High resilience of item representation to sensory inference and oro-sensory disturbances

Recognition accuracy was well above chance at all levels of disturbance (Fig. 3a). The accuracy was highest on trials with a single disturbance and exhibited only small decrements with each additional disturbance (repeated measures analysis of variance, rmANOVA: F_4,80_ = 6.25, p < 0.001, η_p_^2^ = 0.23). Relative to trials with one disturbance (M = 82%, SD = 6.38%), the accuracy was reduced by only 2% on trials with two disturbances (M = 80%, SD = 5.22%; t_20_ = 1.56, d = 0.34, p_bonf_ = 1, 95% CI = [− 0.019, 0.056]), by 5% on trials with three disturbances (M = 77%, SD = 5.78%; t_20_ = 4.15, d = 0.91, p_bonf_ = 0.005, 95% CI = [0.013, 0.091]), and by 6% on trials with four disturbances (M = 76%, SD = 6.6%; t_20_ = 3.61, d = 0.79, p_bonf_ = 0.017, 95% CI = [0.008, 0.120]). Accuracy was lower when the probe was presented immediately after the taste than when presented after a single disturbance (M = 78%, SD = 7.70%; t_20_ = − 3.25, d = − 0.71, p_bonf_ = 0.04; see Suppl. Table 1 for all pairwise comparisons).

To assess whether participants were aware of their performance, we assessed the link between confidence and accuracy. Confidence about recognition decisions had a strong veridical relationship to the accuracy of those decisions, i.e. trials with a higher confidence rating also had a higher accuracy (F_3,60_ = 60.86, p < 0.001, η_p_^2^ = 0.75; Fig. 3b), indicating that participants indeed monitored their performance.

In summary, the relatively small decrements in recognition accuracy with increasing disturbances cannot be solely accounted for by either (i) attentional distraction/interference (Fig. 2b,d) or (ii) sensory interference in gustatory WM (Fig. 2b,c). However, the lowered accuracy for zero disturbances suggests sensory interference in the sensory encoding of the probe stimulus caused by the temporal proximity of the item and probe. We, therefore, restricted our subsequent analyses to disturbances ≥ 1.

#### Evidence for the representation of an item’s taste rather than other associated properties

We next tested whether taste recognition’s apparent resilience was indeed due to the representation of the item’s taste rather than some other associated property such as stimulus similarity. We exploited the well-known role of stimulus similarity in recognition with other stimulus modalities^33–37^. As a simplistic illustration, a pair of phonetically similar but non-identical words, e.g., ‘cat’ and ‘cap’, might be more likely to be (mis)judged as being “same” than a dissimilar word pair, e.g., ‘cat’ and ‘pig’. A comparable prediction here would be that recognition judgments involving a WM for taste should depend on the chemical similarity of the item-probe pairs, see^15^ for a similar argument. To test this, we compared the recognition accuracy for item-probe foil pairs that shared a chemical component (e.g., item: *sour*, probe: *sour–sweet*) with pairs that did not (e.g., item: *sour*, probe: *bitter*). Item-probe foil pairs with a shared component were assumed to be more similar (intermediate similar) than those without a shared component (lowest in similarity). If this assumption holds, item-probe foil pairs of intermediate similarity should have a higher proportion of “same” responses (i.e., lower accuracy) than those with the lowest similarity. Furthermore, the probability of “same” responses should be the highest for item-probe match pairs, which are chemically identical and perceptually highest in similarity.

In line with these predictions, the proportion of “same” responses varied between item-probe pairs (rmANOVA; F_2,40_ = 516.51, p < 0.001, η_p_^2^ = 0.9267; Fig. 3c) such that the proportion of “same” responses was highest for the item-probe match pairs that were highest in similarity (M = 80.38%, SD = 9.22%; highest similarity versus intermediate in similarity similar: t_20_ = 20.16, p_bonf_ < 0.001, d = 4.4, 95% CI = [0.4017, 0.5213]; intermediate in similarity versus low in similarity: t_20_ = 32.23, p_bonf_ < 0.001, d = 7.03, 95% CI = [0.6315, 0.7429]). Importantly, the proportion of “same” responses for the intermediary similar pairs (M = 34.23%, SD = 11.84%) was significantly greater than for the lowest in similarity pairs (M = 11.66%, SD = 6.96%; t_20_ = 10.68, p_bonf_ < 0.001, d = 2.33, 95% CI = [0.1704, 0.2809]).

In summary, the modulation of recognition by taste similarity relationships is consistent with representing an item’s taste in WM rather than, for example, a verbal label describing the item’s taste.

#### Disturbances have dissociable effects on match and foil trials

The finding of a stimulus-similarity effect provided a way to evaluate why accuracy might reduce with increasing disturbances. Stimulus-similarity suggests that the combination of increasing disturbances and time-dependent forgetting might lower the precision of a taste representation in memory. If so, this loss of precision should reduce the item-probe similarity (and accuracy) to a greater extent on match than foil trials. Consistent with a predicted match/foil dissociation, recognition accuracy on match and foil trials (Fig. 3d) were modulated differently by the number of disturbances (rmANOVA; Disturbances {1, 2, 3, 4} × Trial type {match, foil}; Disturbances × Type: F_3,60_ = 6.70, p < 0.001, η_p_^2^ = 0.26; Disturbances: F_3,60_ = 8.33, p < 0.001, η_p_^2^ = 0.29; Trial type: F_1,20_ = 0.8511, p = 0.3672, η_p_^2^ = 0.04). While match trials showed an accuracy reduction of 13% with increasing disturbances (F_3,60_ = 17.72, p < 0.001), accuracy on foil trials was not measurably affected by the number of disturbances (F_3,60_ = 0.31, p = 0.82).

Additionally, response times on correct trials (RTs; Fig. 3e) increased with the number of disturbances on match (F_3,60_ = 11.32, p < 0.001; Suppl. Table 2) but not foil (F_3,60_ = 1.63, p = 0.1923; Suppl. Table 2) trials. This resulted in a significant interaction (rmANOVA; Number of disturbances {1,2,3,4} × Trial type {match, foil}, Disturbances × Type: F_3,60_ = 4.64, p < 0.006, η_p_^2^ = 0.19; Disturbances: F_3,60_ = 7.99, p < 0.001, η_p_^2^ = 0.29; Type; F_1,20_ = 3.19, p = 0.089, η_p_^2^ = 0.14).

Furthermore, confidence was significantly lower for match than foil trials (Fig. 3f; rmANOVA; Disturbances {1,2,3,4} × Trial Type {match, foil}; Disturbances × Type: F_3,60_ = 4.46, p = 0.0068, η_p_^2^ = 0.18; Disturbances: F_3,60_ = 10.45, p < 0.001, η_p_^2^ = 0.34; Trial Type: F_1,20_ = 30.97, p < 0.001, η_p_^2^ = 0.61).

Taken together, the observed pattern of results where match trials were characterized by lower accuracy, higher RTs, and lower confidence than foil trials were consistent with a loss in precision of the item’s representation in memory, according to a stimulus similarity rationale.

### Capacity for multiple distinct tastes (Experiment 2)

We next sought to estimate the capacity of taste working memory using a MIR task (Figs. 1a and 2b). Participants were to remember one to five sequentially presented tastes (items) on each trial, representing set sizes of one to five (referred to as S1 to S5). A tasteless stimulus (rinse) followed each item to cleanse sapid residues with minimal taste recognition disturbance to minimize sensory interference. Item memory was then tested with a probe stimulus. Participants indicated whether the probe matched any of the items in the remembered set (i.e., match) or not (i.e., foil) and then rated their confidence in that decision. We sought to assess WM capacity based on how recognition accuracy changed with increasing set size.

#### Non-uniform reduction in recognition accuracy with increasing set size

Recognition accuracy decreased monotonically with increasing set size (rmANOVA: F_4,76_ = 37.52, p < 0.001, η_p_^2^ = 0.66; Fig. 4a). Accuracy was highest for S1 (M = 73.2%, SD = 10.8%). Although each additional increase in set size lowered accuracy, the relative decrements in accuracy for each additional item was not uniform. The decrement was 8.9% for S2 relative to S1 (t_20_ = 4.93, d = 1.10, p_bonf_ < 0.001, 95% CI = [0.023, 0.108]) and 7.9% for S3 relative to S2 (t_20_ = 3.71, d = 0.83, p_bonf_ = 0.015, 95% CI = [0.008, 0.098]). However, each additional increase in set size beyond three produced smaller decrements, namely, 3.1% for S4 relative to S3 (t_20_ = 1.41, d = 0.32, pbf = 1, 95% CI = [− 0.024, 0.063]) and 5.4% for S5 relative to S4 (t_20_ = 2.13, d = 0.48, p_bonf_ = 0.469, 95% CI = [− 0.016, 0.089]; see Suppl. Table 3 for full pairwise comparisons). This non-uniform change was further supported by the qualitatively steeper negative slope fitting S1, S2, and S3 (Fig. 4a; − 0.059) compared to the slope fitting S3, S4, and S5 (Fig. 4a; − 0.026).

We next sought to disentangle the relative contributions of the match and foil trials to the observed reductions in recognition accuracy with set size increases.

#### Larger accuracy reductions for foil versus match trials with increasing set size

The match accuracy for a particular set-size was the mean match accuracy across all possible probe-item configurations for that set size (e.g., for set size 2, the probe could match item 1 or item 2).

A key consideration when evaluating the effect of set-size on match and foil accuracy was the role of stimulus-similarity. In the SIR task (Experiment 1), recognition accuracy for similar item-probe foil pairs was lower than for dissimilar pairs (Fig. 3c). Accounting for the equivalent effects of stimulus-similarity with the multi-item sets was crucial since stimulus-similarity depends on the type of tastes that must be remembered rather than constraints on the number of items that can be remembered (i.e., capacity). Specifically, for multi-item sets, the probability that a probe stimulus shares a taste component with one or more items increases with set size. This could lead to increased errors on foil trials with increasing set size and, conversely, inflate the accuracy of match trials.

Consistent with these hypothesized effects, trial type was a significant modulator of how recognition accuracy changed with set size (rmANOVA: Type {match, foil} × Set size {1,2,3,4,5}, Type × Set size: F_4,76_ = 4.19, p = 0.027, η_p_^2^ = 0.18; Type: F_1,19_ = 9.58, p = 0.006, η_p_^2^ = 0.34; Set size: F_4,76_ = 37.99, p < 0.001, η_p_^2^ = 0.67; Fig. 4b). Accuracy on foil trials decreased by 35.9% with a set size increase from one to five (F_4,76_ = 25.72, p < 0.001). The corresponding effect of set size on match trial accuracy was only 10.8% and not statistically significant (F_4,76_ = 2.21, p = 0.08).

These results show that the reduction in recognition accuracy with increasing set size (Fig. 4a) was dominated by the role of foil trials rather than match trials. Due to the potentially significant role of stimulus-similarity effects when interpreting set-level foil and match accuracy, we focused on match trials of individual items to identify capacity constraints on individual set items.

#### Maximum capacity limit of 3 items

With capacity constraints, an accuracy reduction with increasing set size (Fig. 4a) might be expected. However, these constraints could be organized in different ways. For instance, the hypothesized capacity constraints might have an ensemble effect where all items in the set might be recognized with similar accuracy, but this overall accuracy might reduce with increasing set size^19–21^. Alternatively, capacity constraints might be organized in an all-or-nothing manner where certain items in the set might be recognized with high accuracy while other items might be lost from memory due to capacity constraints^23^.

An important factor in distinguishing these constraints was the sequential presentation of items due to which each item is maintained in WM for differing latencies relative to the probe. To equate inter-item latency differences across set sizes, Fig. 4c shows the recognition (match) accuracy for items at different serial positions in each set relative to the probe. For each set size, recognition accuracy across serial positions varied significantly (one-way rmANOVA; S_2_: F_1,19_ = 28.52, p_bonf_ < 0.001, η_p_^2^ = 0.60; S_3_: F_2,38_ = 15.76, p < 0.001, η_p_^2^ = 0.45; S_4_: F_3,57_ = 10.72, p < 0.001, η_p_^2^ = 0.36; S_5_: F_4,76_ = 9.72, p < 0.001, η_p_^2^ = 0.34). There was a notable recency effect where accuracy was highest for the last item in all set sizes (i.e., with the lowest latency to the probe). Recognition accuracy decreased as the latency between the item and probe increased. Furthermore, a primacy effect was observable for the set sizes 3 and 4.

The prominent recency effect across set sizes also suggests a limited role for sensory adaptation, which would predict increases in encoding interference for tastes presented later in the sequence (Fig. 2).

Importantly, the variable accuracy across items and the comparably high recency effect across set sizes was inconsistent with an ensemble effect, i.e., on average, a relatively uniform decrease in accuracy across all items with increasing set size. To assess whether all the individual set items were held in memory, we tested the accuracy at each serial position against random chance (50%) (Suppl. Table 4). All items showed above-chance accuracy with the critical exception of items with a latency at − 3 and − 4 relative to the probe (Fig. 4c). Specifically, these items were item 2 (abbreviated as I2) in S4 (latency at − 3) and items I2 and I3 in S5 (latency at − 3 and − 4, respectively). Thus, a maximum of only three items could be successfully retained even when the set size required more items to be remembered.

We next assessed whether participants were subjectively aware of this capacity limit on the number of remembered items.

#### Limited subjective awareness of capacity limits

To assess participants’ awareness, we first evaluated RTs (on correct trials) as a possible indicator of recognition uncertainty. Mirroring the effects of trial-type on accuracy (Fig. 4b), trial type also modulated how the mean RTs on correct trials changed with set size (Fig. 5a; rmANOVA; Type {match, foil} × Set size {1,2,3,4,5}; Set size × Type: F_4,76_ = 5.47, p = 0.003, η_p_^2^ = 0.22; Type: F_1,19_ = 7.76, p = 0.012, η_p_^2^ = 0.29; Set size: F_4,76_ = 1.16, p = 0.326, η_p_^2^ = 0.06;). Foil RTs increased with set size by as much as 136.7 ms from set size one to five (F_4_ = 4.03, p = 0.005), while the corresponding change in match RTs was not statistically significant (F_4_ = 1.39, p = 0.25). This similarity in RTs across match trials was notable since the set sizes S4 and S5 were affected by capacity limits on the number of remembered items. A more direct indicator of subjective awareness was provided by how participants rated their confidence in their response on each trial. As in Experiment 1, trials with a higher confidence rating also had higher accuracy in Experiment 2 (Fig. 5b; rmANOVA; F_4,46_ = 6.95, p = 0.004, η_p_^2^ = 0.27). However, when separated by trial type (i.e., match and foil), confidence on correct trials changed with set size (Fig. 5c; rmANOVA; Type {match, foil} × Set size {1,2,3,4,5}; Type × Set size: F_4,76_ = 12.85, p < 0.001, η_p_^2^ = 0.404; Type: F_1,19_ = 0.09, p = 0.766, η_p_^2^ = 0.005; Set size: F_4,76_ = 24.667, p < 0.001, η_p_^2^ = 0.565). Resolving the interaction via simple main effects analyses, we found a reduction in confidence with increasing set size for foil trials (F_4,76_ = 29.44, p < 0.001; see Suppl. Table 5 for pairwise tests). However, confidence in match trials remained largely unchanged by set size. Although the match trials exhibited a significant influence of set size (F_4,76_ = 5.07, p = 0.001 ), except for a single pair, none of the other pairwise differences were statistically significant (Suppl. Table 5).

Taken together, the modulation of the RTs and confidence ratings on foil trials but not match trials mirrored the dissociation in recognition accuracy (Fig. 4b). As stimulus-similarity effects rather than capacity limitations putatively drove this dissociation of match/foil accuracy effects, this finding suggests that the RTs and confidence ratings might be indicators of the subjective uncertainty arising from stimulus-similarity and not about capacity per se.

## Discussion

We used two novel single and multi-item taste recognition tasks to investigate gustatory WM’s organization and capacity limit. Participants reliably recalled single items despite oro-sensory distractions suggesting that tastes were actively and resiliently maintained. Participants distinctly and reliably maintained up to three tastes for subsequent recognition with varying resolution when multiple tastes were presented. This shows that the concept of WM extends to perceptual information from the gustatory system.

### Resilient representation of single taste items

WM capacity requires attention^2,38^ which can be exhausted by various aspects of the task unrelated to WM. In a previous study of gustatory WM^15^, the presentation of each taste stimulus involved three steps: participants were to grasp a cup with the stimulus liquid, take a sip, and then spit it out. We used an automated system to deliver the taste stimuli to avoid the detrimental effects on WM of such multiple tasks^39^. Nevertheless, the oro-sensory stimulation associated with a taste stimulus was a potential disturbance that could take up attentional resources and affect WM maintenance. In our test of this possibility (Experiment 1), we found that recognition accuracy for a single taste to be well above chance and only marginally influenced by the presence of multiple tasteless oro-sensory disturbances. Although recognition accuracy decreased with each additional disturbance, and hence also with increasing time, these decrements were relatively small (2–6%), suggesting that attentional interference and time-based forgetting played only minor roles.

Sensory interference from residues of prior stimuli or adaptation could have impeded the sensory encoding of subsequent stimuli^26,40^ and hence lowered recognition accuracy independent of capacity limitations, particularly for the short intervals between consecutive taste presentations. Our results indicate a limited role for both these factors. In Experiment 1, item-probe pairs separated by a single tasteless stimulus had a slightly higher recognition accuracy as compared to pairs that were not (Fig. 3a). This might be because the tasteless stimulus successfully removed the items’ residuals before probe presentation. We thus presented a tasteless stimulus after each taste in Experiment 2 to serve as a rinse. If adaptation had a prominent role (as hypothesized in Fig. 2c), recognition accuracy in Experiment 1 would have been expected to increase with the increasing time between item and probe as adaptation’s detrimental effects faded. However, for the sequential presented items in Experiment 2, adaptation would have been expected to have a more considerable impact on items presented later rather than earlier during the sequence. Neither of these predicted effects was evident in our results. Although we cannot exclude (cross)adaptation in Experiment 2, its effects were likely ineffectual because the tastes were adequately encoded and maintained for later retrieval. Furthermore, accuracy was highest for the last item in a set (recency effect; Fig. 4c), which is incompatible with habituation. However, we cannot exclude that a release of adaptation compensates for detrimental effects of attentional interference.

Another possible source of encoding-related distortions was crossmodal cueing, including verbalized encoding of the taste qualities. Participants were instructed not to name the stimuli and confirmed this during debriefing. Our results suggest participants’ compliance: recognition accuracy was systematically modulated by the chemosensory similarity between stimuli, which is incompatible with a purely verbal encoding of the stimuli. Furthermore, the reduction in match accuracy with increasing disturbances in Experiment 1 cannot be explained with the ease of remembering a single word over extended periods.

### Stimulus similarity assumptions

We used mixtures to expand the gustatory stimulus space beyond basic tastes. It is hypothetically possible that a mixture might take up more memory than a simple taste and affect performance if a feature-based organization of WM is assumed, which is notably debated in the visual domain^22^, but see^41^. We assumed that stimulus complexity would have only a minor effect, if any, because the fast pace and high task-demands in our study precluded an analytic strategy to identify the mixture components and informal reports of participants confirmed this. If it had an effect, it would be an underestimation of WM capacity, in line with observations in visual WM^22^.

**Figure 6:**
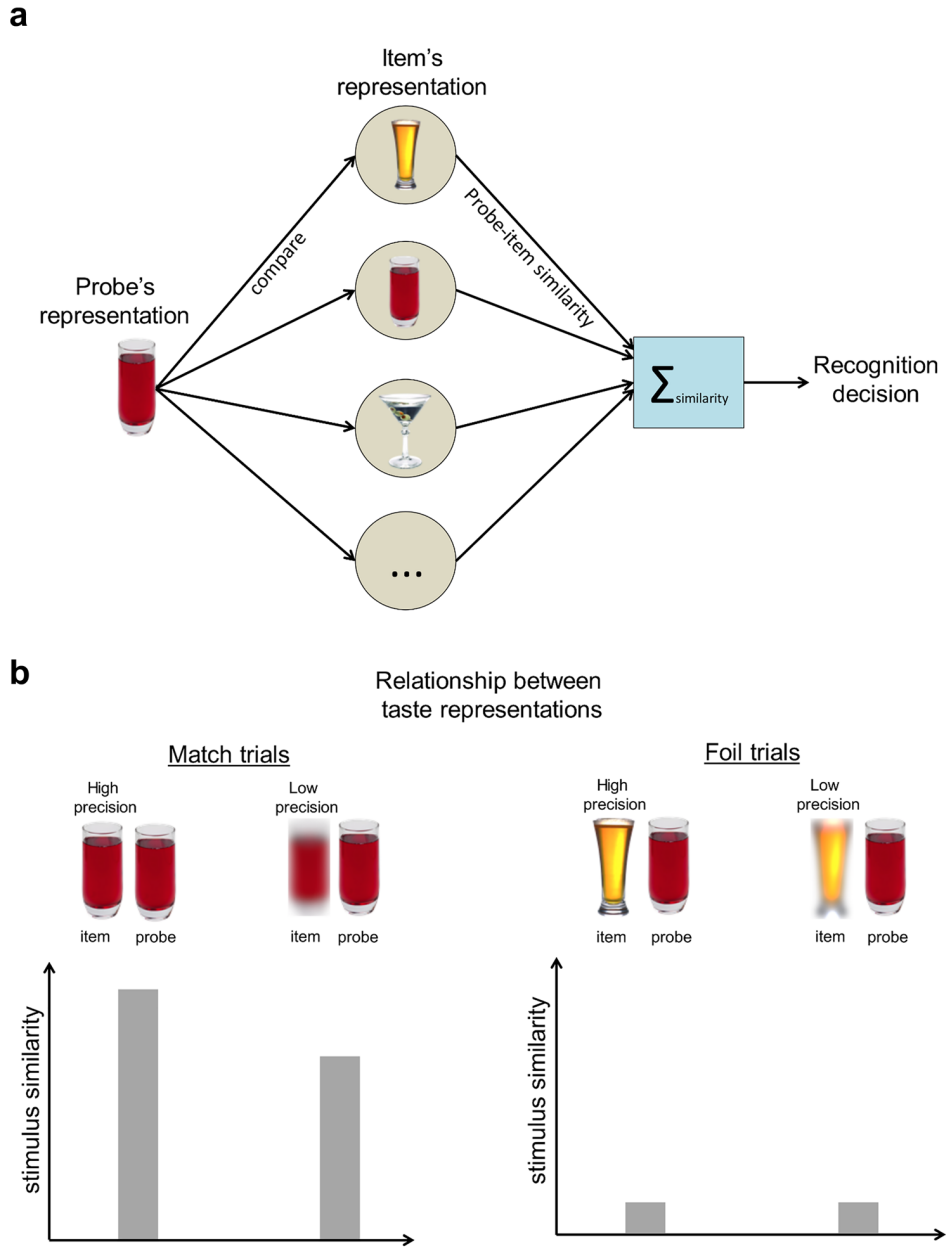
Stimulus similarity. (**a**) When the probe is compared to each item in a set, this comparison estimates these representations’ similarities. These similarity values are then summed together and compared against a decision criterion. (**b**) Left panel: for the match trials, when the item has high precision, there should be a high similarity to the probe’s representation. Conversely, the relative reduction in the precision of the item’s representation should lower the probe-item similarity. Right panel: for the foil trials, it is assumed that the probe-item similarity might already be low. Hence, any reductions in the precision might not further lower this similarity.

We use the qualitative predictions of computational models of stimulus-similarity on recognition to guide the interpretation of our findings. The rationale is based on computational models that characterize the critical role of stimulus similarity in recognition. Our focus is one influential family of models (the Generalized Context Model^42^, Noisy Exemplar Model^34,43^, where recognition is assumed to involve a comparison of the probe to each of the items in a set in memory as illustrated in Fig. 6a. According to these models, each probe-item comparison produces an estimate of the similarity of these representations. The similarity values are then summed together and compared against a decision-criterion. These models are grounded in the theoretical signal detection view of how the summed similarity is converted into a decision^34,43^. We used the qualitative predictions of these stimulus similarity-based models to guide the interpretation of our findings.

Based on the above stimulus-similarity rationale, the loss in precision in an item’s representation might be expected to have a more significant effect on match trials than foil trials (see Fig. 6b). The left panel depicts the assumed effect of precision changes on match trials. When the item has high precision, there should be a high similarity to the probe representation. Hence, a relative reduction in the precision of this representation should lower the probe-item similarity. However, for the foil trials (right panel), we assume that the probe-item similarity might already be low. Hence, any reductions in the precision might not further lower this similarity. This rationale accounts for our findings in Experiment 1, where match trials show a more significant reduction in accuracy with increasing disturbances than foil trials.

In Experiment 1 and 2, participants did not know in advance whether a trial would be a match or lure trial. Therefore, we assume that the decision criterion is the same for match/foil trials. However, it is theoretically possible that the number of disturbances (Experiment 1) or the set-size (Experiment 2) could be used as a cue for an online adjustment of the decision-criterion^34,43–46^. Experiment 2 suggests that this might not have been the case since the accuracy of the final items’ recognition was similar across set sizes (Fig. 4c).

### Performance monitoring

Since WM has been traditionally proposed to represent consciously perceived information^27,33,47^, we tested whether participants were aware of their memory performance through retrospective confidence ratings. Metacognitive judgments such as confidence ratings have been proposed to measure performance monitoring and perceptual awareness and to track accuracy in numerous, albeit not all, visual WM tasks^48,49^. In both experiments, confidence tracked overall recognition accuracy, such that trials with higher confidence had a higher accuracy indicating that participants were aware of the tastes (Figs. 3b, 5b). These findings align well with previous visual WM observations, where metacognitive awareness tracked recognition accuracy, suggesting that participants monitor their performance, crucial for WM success^48^.

However, recognition accuracy in our experiments had another important determinant apart from capacity, namely, stimulus-similarity. The dissociation between match and foil trials in both experiments suggests that the confidence ratings and RTs (which can be viewed as an index of decision difficulty) were indicators of the subjective uncertainty related to stimulus-similarity rather than an awareness of capacity limitations.

In Experiment 1, the accuracy on match trials showed a greater modulation by the number of disturbances than on foil trials (Fig. 3d), consistent with a reduction in item-probe similarity as the item’s memory representation degraded. Following this pattern, the confidence ratings were lower for match trials (Fig. 3f), and RTs were higher for match trials and increased with the number of disturbances, unlike the foil trials (Fig. 3e).

This accuracy-confidence link was inverted in Experiment 2, where the foil trials exhibited a substantially larger reduction in accuracy with set-size than the match trials (Fig. 4b). Even though a probe might be weakly similar to any single item, the cumulative similarity of the probe to the set was higher as set size increased, potentially leading to increased misjudgments of foil trials. Again, following this pattern, the confidence ratings for foil trials showed a greater modulation by set size than on match trials (Fig. 5c), and the RTs for foil trials were larger and modulated to a greater extent by set size than match trials (Fig. 5a). It was especially notable that participants seemingly failed to track that they "dropped" one or more items in the larger sets.

Together, our results suggest a subjective awareness of similarity-related uncertainty but not necessarily an awareness of capacity limitations.

### Storage capacity of gustatory WM

The observed monotonic decrease in recognition accuracy with increasing set size in Experiment 2 is in line with findings from other modalities^2,5,23^ and could be interpreted as an indicator for a limited gustatory WM capacity. However, a set-size-dependent reduction of accuracy is insufficient to unequivocally deduce whether shared-resources constrain working memory^19,21^ or a slot-based fixed item limit^22,23^. We examined recognition accuracy at various positions within the sets to address this question. While most items were recognized with above-chance accuracy, this was not the case for some items in sets larger than 3, indicating that these items were lost from memory due to capacity constraints. The observed serial-position effects also show that some items are represented at a better resolution than others. This is incompatible with an ensemble effect, which predicts that cognitive resources, on average, to be shared equally among items in a set^19–21^. Instead, a slot-based limited item model, according to which the maximum number of stimuli is discretely and with adequate resolution represented^22,23,50,51^, better explains the observed WM performance. It is also conceivable that our results reflect the loss of unattended information in gustatory WM. If each new item reduces and eventually exhausts the available attentional resources, unattended items may be degraded to an extent where recognition is rendered impossible.

Furthermore, we found that trial type (match or foil) was a significant modulator of how recognition accuracy changed with set size, such that the set-size dependent accuracy decrease was primarily driven by foil and not match trials. This variation is not readily explained by a slot-based limited item model, which would predict a comparable performance on foil and match trials. Instead, stimulus-similarity has most likely contributed to, if not caused, the set-size-dependent accuracy decrease because it is more likely to occur in larger sets and affect foil trials more than match trials. However, stimulus-similarity would be affected by the resolution of representations rather than the number of items that can be remembered^50,52,53^. Taken together, our results advocate a hybrid model of gustatory WM with a limited number of slots where items are stored with varying amounts of precision^52^, for similar argument see^53^.

## Supplementary Information

Supplementary Information.
