# Supplementary material for: The capacity and organization of gustatory working memory
Source: Sci Rep. 2022 May 16;12:8056. doi: 10.1038/s41598-022-12005-x (PMC9110745; doi:10.1038/s41598-022-12005-x)
Supplement: Supplementary file 1 — Supplementary Information. [file 41598_2022_12005_MOESM1_ESM.docx]

Lim et al. The Capacity and Organization of Gustatory Working Memory

**Supplementary Material**


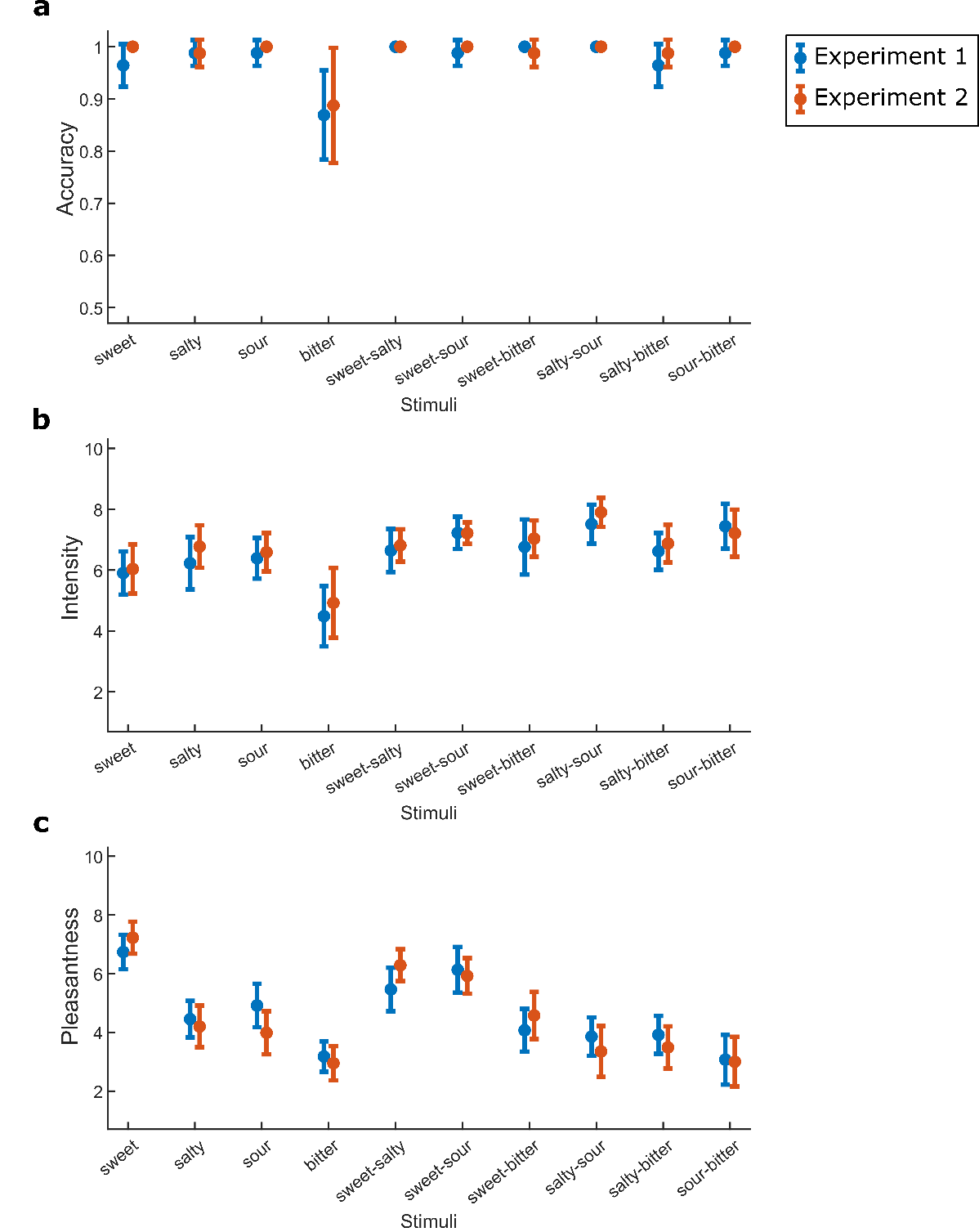


**Supplementary Fig. 1 | Taste Detection and Evaluation Task (TDE). a,** Taste detection accuracy (proportion correct), **b,** intensity ratings (1=not intense, 10=very intense), and **c,** pleasantness ratings (1=not pleasant, 10=very pleasant) for each of the ten stimuli. Data points are means, error bars are SEM. N=21 for Experiment 1 (blue) and N=20 for Experiment 2 (red).

**Supplementary Table 1 |** Pairwise comparisons of accuracy for different numbers of disturbances (0-4) in Experiment 1.

| **Pairwise Comparisons - number of disturbance** | | | | | | | | |
| --- | --- | --- | --- | --- | --- | --- | --- | --- |
|  | | **95% CI for Mean Difference** | |  | | | | |
| **Pairs (number of disturbances)** | | **Lower** | **Upper** | **SE** | **t** | **Cohen's d** | **p_bonf_** | |
| 0 | 1 | -0.0898 | -0.0013 | 0.0140 | -3.2476 | -0.7087 | 0.0403 | * |
|  | 2 | -0.0699 | 0.0160 | 0.0136 | -1.9780 | -0.4316 | 0.6187 |  |
|  | 3 | -0.0396 | 0.0522 | 0.0145 | 0.4336 | 0.0946 | 1.0000 |  |
|  | 4 | -0.0395 | 0.0769 | 0.0185 | 1.0125 | 0.2209 | 1.0000 |  |
| 1 | 2 | -0.0191 | 0.0564 | 0.0120 | 1.5585 | 0.3401 | 1.0000 |  |
|  | 3 | 0.0125 | 0.0913 | 0.0125 | 4.1526 | 0.9062 | 0.0049 | ** |
|  | 4 | 0.0082 | 0.1204 | 0.0178 | 3.6139 | 0.7886 | 0.0173 | * |
| 2 | 3 | -0.0064 | 0.0729 | 0.0126 | 2.6437 | 0.5769 | 0.1558 |  |
|  | 4 | -0.0064 | 0.0976 | 0.0165 | 2.7660 | 0.6036 | 0.1192 |  |
| 3 | 4 | -0.0337 | 0.0584 | 0.0146 | 0.8482 | 0.1851 | 1.0000 |  |
|  | | | | | | | | |
| * p < .05, ** p < .01 | | | | | | | | |
| *Note.*  Cohen's d does not correct for multiple comparisons. | | | | | | | | |
| *Note.*  P-values and confidence intervals are adjusted for comparing a family of 10 estimates (confidence intervals are corrected using the Bonferroni method). | | | | | | | | |

**Supplementary Table 2 |** Pairwise comparisons of accuracy for different numbers of disturbances (0-4) for match and lure trials in Experiment 1.

| **Pairwise Comparisons (match trials)** | | | | | | | | |
| --- | --- | --- | --- | --- | --- | --- | --- | --- |
|  | | **95% CI for Mean Difference** | |  | | | | |
| **Pairs (number of disturbances)** | | **Lower** | **Upper** | **SE** | **t** | **Cohen's d** | **p_bonf_** | |
| 1 | 2 | 0.0023 | 0.0873 | 0.0145 | 3.0853 | 0.6733 | 0.0350 | * |
|  | 3 | 0.0374 | 0.1434 | 0.0181 | 4.9888 | 1.0887 | < .001 | *** |
|  | 4 | 0.0607 | 0.2064 | 0.0249 | 5.3676 | 1.1713 | < .001 | *** |
| 2 | 3 | -0.0041 | 0.0952 | 0.0170 | 2.6871 | 0.5864 | 0.0850 |  |
|  | 4 | 0.0187 | 0.1588 | 0.0239 | 3.7074 | 0.8090 | 0.0084 | ** |
| 3 | 4 | -0.0011 | 0.0875 | 0.0151 | 2.8528 | 0.6225 | 0.0590 |  |
| **Pairwise Comparisons (lure trials)** | | | | | | | | |
| 1 | 2 | -0.0567 | 0.0425 | 0.0170 | -0.4181 | -0.0912 | 1.0000 |  |
|  | 3 | -0.0545 | 0.0812 | 0.0232 | 0.5750 | 0.1255 | 1.0000 |  |
|  | 4 | -0.0761 | 0.0670 | 0.0244 | -0.1856 | -0.0405 | 1.0000 |  |
| 2 | 3 | -0.0558 | 0.0967 | 0.0261 | 0.7840 | 0.1711 | 1.0000 |  |
|  | 4 | -0.0622 | 0.0673 | 0.0221 | 0.1154 | 0.0252 | 1.0000 |  |
| 3 | 4 | -0.0919 | 0.0562 | 0.0253 | -0.7066 | -0.1542 | 1.0000 |  |
| * p < .05, ** p < .01, *** p < .001 | | | | | | | | |
| *Note.*  Cohen's d does not correct for multiple comparisons. | | | | | | | | |
| *Note.*  P-values and confidence intervals are adjusted for comparing a family of 6 estimates (confidence intervals are corrected using the Bonferroni method). | | | | | | | | |

**Supplementary Table 3 |** Pairwise comparisons of accuracy of accuracy for different set sizes (1-5) in Experiment 2.

| **Pairwise Comparisons - set size** | | | | | | | | |
| --- | --- | --- | --- | --- | --- | --- | --- | --- |
|  | | **95% CI for Mean Difference** | |  | | | | |
| **Pairs (set size)** | | **Lower** | **Upper** | **SE** | **t** | **Cohen's d** | **p_bonf_** | |
| 1 | 2 | 0.0233 | 0.1075 | 0.0133 | 4.9289 | 1.1021 | < .001 | *** |
|  | 3 | 0.0656 | 0.1705 | 0.0165 | 7.1483 | 1.5984 | < .001 | *** |
|  | 4 | 0.0854 | 0.1888 | 0.0163 | 8.4204 | 1.8828 | < .001 | *** |
|  | 5 | 0.1012 | 0.2372 | 0.0214 | 7.8950 | 1.7654 | < .001 | *** |
| 2 | 3 | 0.0076 | 0.0977 | 0.0142 | 3.7092 | 0.8294 | 0.0149 | * |
|  | 4 | 0.0326 | 0.1109 | 0.0123 | 5.8171 | 1.3008 | < .001 | *** |
|  | 5 | 0.0511 | 0.1565 | 0.0166 | 6.2537 | 1.3984 | < .001 | *** |
| 3 | 4 | -0.0238 | 0.0619 | 0.0135 | 1.4134 | 0.3161 | 1.0000 |  |
|  | 5 | 0.0133 | 0.0890 | 0.0119 | 4.2872 | 0.9587 | 0.0040 | ** |
| 4 | 5 | -0.0158 | 0.0799 | 0.0151 | 2.1250 | 0.4752 | 0.4693 |  |
| * p < .05, ** p < .01, *** p < .001 | | | | | | | | |
| *Note.*  Cohen's d does not correct for multiple comparisons. | | | | | | | | |
| *Note.*  P-values and confidence intervals are adjusted for comparing a family of 10 estimates (confidence intervals are corrected using the Bonferroni method). | | | | | | | | |

**Supplementary Table 4 |** One sample t-tests comparing the accuracy for each position in a set to chance level.

| **One Sample T-Test** | | | | | | |
| --- | --- | --- | --- | --- | --- | --- |
| **set size** | **position from probe** | **t** | **df** | **p** | **p_bonf_** | **Cohen's d** |
| 5 | -5 | 2.602 | 19 | 0.0088 | 0.044 | 0.5818 |
|  | -4 | 1.4405 | 19 | 0.083 | 0.415 | 0.3221 |
|  | -3 | 2.0945 | 19 | 0.0249 | 0.1245 | 0.4684 |
|  | -2 | 8.0264 | 19 | < .001 | < 0.005 | 1.7948 |
|  | -1 | 7.9167 | 19 | < .001 | < 0.005 | 1.7702 |
| 4 | -4 | 4.1436 | 19 | < .001 | < 0.004 | 0.9265 |
|  | -3 | 1.8827 | 19 | 0.0376 | 0.1504 | 0.421 |
|  | -2 | 3.5652 | 19 | 0.001 | 0.004 | 0.7972 |
|  | -1 | 8.9532 | 19 | < .001 | < 0.004 | 2.002 |
| 3 | -3 | 4.0228 | 19 | < .001 | <0.003 | 0.8995 |
|  | -2 | 4.568 | 19 | < .001 | <0.003 | 1.0214 |
|  | -1 | 11.0101 | 19 | < .001 | <0.003 | 2.4619 |

**Supplementary Table 5 |** Pairwise comparisons of accuracy of accuracy for different set sizes (1-5) for match and lure trials in Experiment 2.

| **Pairwise Comparisons (match trials)** | | | | | | | | |
| --- | --- | --- | --- | --- | --- | --- | --- | --- |
|  | | **95% CI for Mean Difference** | |  | | | | |
| **Pairs (set size)** | | **Lower** | **Upper** | **SE** | **t** | **Cohen's d** | **p_bonf_** | |
| 1 | 2 | 0.0305 | 0.3499 | 0.0503 | 3.7788 | 0.8450 | 0.0127 | * |
|  | 3 | -0.0529 | 0.3614 | 0.0653 | 2.3638 | 0.5286 | 0.2889 |  |
|  | 4 | -0.0056 | 0.3843 | 0.0614 | 3.0819 | 0.6891 | 0.0614 |  |
|  | 5 | -0.0161 | 0.4227 | 0.0691 | 2.9412 | 0.6577 | 0.0838 |  |
| 2 | 3 | -0.1901 | 0.1183 | 0.0486 | -0.7391 | -0.1653 | 1.0000 |  |
|  | 4 | -0.1183 | 0.1166 | 0.0370 | -0.0237 | -0.0053 | 1.0000 |  |
|  | 5 | -0.1393 | 0.1655 | 0.0480 | 0.2730 | 0.0611 | 1.0000 |  |
| 3 | 4 | -0.0637 | 0.1337 | 0.0311 | 1.1261 | 0.2518 | 1.0000 |  |
|  | 5 | -0.1423 | 0.2403 | 0.0603 | 0.8130 | 0.1818 | 1.0000 |  |
| 4 | 5 | -0.1324 | 0.1604 | 0.0461 | 0.3032 | 0.0678 | 1.0000 |  |
| **Pairwise Comparisons (lure trials)** | | | | | | | | |
| 1 | 2 | 0.0963 | 0.4590 | 0.0571 | 4.8586 | 1.0864 | 0.0011 | ** |
|  | 3 | 0.2119 | 0.6365 | 0.0669 | 6.3421 | 1.4181 | < .001 | *** |
|  | 4 | 0.2288 | 0.7495 | 0.0820 | 5.9623 | 1.3332 | < .001 | *** |
|  | 5 | 0.4094 | 0.9725 | 0.0887 | 7.7887 | 1.7416 | < .001 | *** |
| 2 | 3 | -0.0087 | 0.3019 | 0.0489 | 2.9954 | 0.6698 | 0.0744 |  |
|  | 4 | 0.0526 | 0.3703 | 0.0501 | 4.2249 | 0.9447 | 0.0046 | ** |
|  | 5 | 0.1854 | 0.6411 | 0.0718 | 5.7565 | 1.2872 | < .001 | *** |
| 3 | 4 | -0.1263 | 0.2560 | 0.0602 | 1.0769 | 0.2408 | 1.0000 |  |
|  | 5 | 0.0549 | 0.4784 | 0.0667 | 3.9968 | 0.8937 | 0.0077 | ** |
| 4 | 5 | -0.0131 | 0.4168 | 0.0677 | 2.9797 | 0.6663 | 0.0770 |  |
| * p < .05, ** p < .01, *** p < .001 | | | | | | | | |
| *Note.*  Cohen's d does not correct for multiple comparisons. | | | | | | | | |
| *Note.*  P-values and confidence intervals are adjusted for comparing a family of 10 estimates (confidence intervals are corrected using the Bonferroni method). | | | | | | | | |
